# Enhanced photocatalytic activity of green synthesized zinc oxide nanoparticles using low-cost plant extracts

**DOI:** 10.1038/s41598-024-66975-1

**Published:** 2024-07-19

**Authors:** Sahar A. Mousa, D. A. Wissa, H. H. Hassan, A. A. Ebnalwaled, S. A. Khairy

**Affiliations:** 1 Physics Department, Faculty of Science, Cairo University, Giza, Egypt; 2 Solid State Physics Department, Physics Research Institute, National Research Centre, Giza, Egypt; 3 Electronics & Nano Devices Lab, Physics Department, Faculty of Science, South Valley University, Qena, 83523 Egypt

**Keywords:** Plant extract, ZnO NPs, Photodegradation, Oxygen vacancies, Green synthesis, Methyl orange (MO), Photocatalytic properties, Materials science, Physics

## Abstract

Developing stable and highly efficient metal oxide photocatalysts remains a significant challenge in managing organic pollutants. In this study, zinc oxide nanoparticles (ZnO NPs) were successfully synthesized using various plant extracts, pomegranate (P.M), beetroot roots (B.S), and seder, along with a chemical process. The produced ZnO NPs were characterized using X-ray diffraction (XRD), Fourier-transform infrared spectroscopy (FT-IR), ultraviolet–visible spectroscopy (UV–Vis), Field Emission Scanning Electron Microscope (FESEM), High-Resolution Transmission Electron Microscopy (HRTEM), and Surface Area. For all prepared samples, the results indicated that the composition of the plant extract affects several characteristics of the produced particles, such as their photocatalytic properties, energy bandgap (E_g_), particle size, and the ratio of the two intensity (0 0 2) and (1 0 0) crystalline planes. The particle size of the produced NPs varies between 20 and 30 nm. To examine NPs' photocatalytic activity in the presence of UV light, Methyl Orange (MO) was utilized. The E_g_ of  ZnO synthesized by the chemical method was 3.16 e. V, whereas it was 2.84, 2.63, and 2.59 for P.M, Seder, and B.S extracts, respectively. The most effective ZnO NPs, synthesized using Beetroots, exhibited a degradation efficiency of 87 ± 0.5% with a kinetic rate constant of 0.007 min^−1^. The ratio of the two intensity (0 0 2) and (1 0 0) crystalline planes was also examined to determine a specific orientation in (0 0 2) that is linked to the production of oxygen vacancies in ZnO, which enhances their photocatalytic efficiency. Furthermore, the increase in photocatalytic effectiveness can be attributed to the improved light absorption by the inter-band gap states and effective charge transfer.

## Introduction

The global industrialization process has greatly enhanced the industrial and social conditions of contemporary life. However, a bigger contributing aspect to sustainable growth is, in fact, environmental protection challenges^1^. Organic compounds, a significant source of environmental contamination, are one of the most harmful pollutants in the water. Due to their toxicity, even at low levels, these substances pose a substantial threat to human health. Additionally, they are often non-biodegradable due to their high stability to light and oxidation, making their degradation process extremely challenging. Consequently, the elimination of hazardous organic dyes from industrial waste is a significant ecological concern. Research in the field of pollutant treatment is focused on the reproduction of highly reactive O_2_⋅ and OH⋅ free radicals to create oxidation processes. Photocatalysis with heterogeneous semiconductors is a highly promising technology^2,3^.

The mechanism of organic molecule degradation utilizing Zinc oxide can be summarized as follows, based on numerous previous observations. The techniques encompass oxidation and reduction, in addition to the generation of electron–hole pairs in the presence of UV radiation. The organic compound is further degraded into CO_2_ and H_2_O, which causes the donor molecule to be oxidized by photoinduced holes and adsorbed oxygen to be reduced by an electron in the oxidation and reduction processes.

Metal oxides can be utilized in a variety of applications due to both their hydrophobic nature and their high energy band gap (e.g., solar cell manufacturing, sensors, and photocatalysis). Furthermore, metal oxides such as TiO_2_, ZnO, and SnO_2_ are utilized as photocatalytic materials for organic pollutant remediation^4^. Plant-based NPs production methods make them popular due to their environmentally friendly methodology. Plant metabolites, such as alkaloids terpenoids, flavonoids, amino acids, enzymes, vitamins, proteins, glycosides, and phenolic chemicals, work well as stabilizing and reducing agents^5–11^. In addition, the nanoparticles produced through plants are more varied in shape and size in comparison with those produced by other organisms such as bacteria, fungi, and algae^12^.

Zinc oxide represents one of the metal oxides used in its nano-size because of its exceptional electrical and optical characteristics, making it suitable for various technological applications. They are characterized by a wide band gap (about 3.37 eV) and high excitation energy (60 MeV)^13,14^. Zinc oxide has a better photocatalytic potential than titanium oxide. Researchers ascribe this gap to the vast number of active sites that effectively produce hydrogen peroxide, increasing the reaction rate^15^.

The synthetic techniques employed to produce nanoparticles are costly and damaging to the environment. As a result, plant-assisted, greener NPs materials are preferred over chemical processes^16^. To eliminate the usage of dangerous substances in the process of synthesizing, biosynthesis is a highly promising approach to produce nanoparticles utilizing cost-effective, ecologically friendly, and biological precursors^17^. Enzymes, plant extracts, and bacteria are three examples of the several biological precursors frequently utilized in the biosynthesis process^18^. The most straightforward method involves utilizing plant extracts to produce NPs due to their abundant availability and cost-effectiveness. Furthermore, the simplicity of expanding plant-based nanoparticle production processes contributes to their popularity, as they employ an environmentally sustainable approach. Plant metabolites, such as alkaloids and phenolic compounds, are effective as reducing agents^12^.

ZnO NPs were synthesized using different chemical methods: hydrothermal^19,20^, microwave-assisted hydrothermal method^21^, Low-temperature co-precipitation process^22^, direct precipitation method^23^, chemical precipitation method^24^, and sol–gel method^25–27^. Green ZnO NPs were also synthesized utilizing several plant extracts as reducing and stabilizing agents. Several varieties of peppers were used (jalapeño, Morita, and ghost)^28^, at different concentrations of citrus macrocarpa extract^29^, shiitake mushroom (Lentinula edodes), and silk sericin extracts^30^, leaf extract solution of Ficus Benjamina L^31^, macrocarpa Monsonia Burkeeana plant extract^32^, Agathosma Betulina extract^14^, Azadirachta India (Neem) leaves extract^33,34^, and Moringaoleifera natural extract^35^.

In this study, ZnO NP photocatalyst is enhanced and prepared using several plant extracts in a straightforward, safe, affordable, and environmentally friendly synthetic methodology that produces a highly effective photocatalyst and lowers toxicity. In this study, nanoparticles were synthesized using extracts derived from three plants: Seder, beetroots, and pomegranate peels. The selection of these types was based on specific criteria, with the most significant factor being their abundance of important compounds (reducing agents) such as flavonoids. These flavonoids are responsible for reducing NPS and are found abundantly in the environment. These agents are considered waste and have a low cost, making them an affordable method to recycle plant waste. Developing stable and highly efficient metal oxide photocatalysts remains a significant challenge in managing organic pollutants. These challenges were limited to using green synthesis to produce ZnO NPs that are both environmentally safe and have enhanced characteristics to employ the green technique. This enhances the photocatalytic activities of the NPs, making them more effective and useful for treating wastewater.

## Experimental

### Materials

No further purification was necessary; all chemicals were used exactly as supplied. MO dye, Zinc acetate dehydrate (CH_3_COO)_2_Zn⋅2H_2_O supplied by Loba India 98%, sodium hydroxide, ethanol, Beetroots, Pomegranate, and Seder leaves.

### Collection of plant material

Following institutional, national, and international requirements, the material collection for Seder, pomegranates, and beetroots was completed. Plant studies and other experimental methods were carried out by relevant institutional, national, and worldwide guidelines. The local market in Egypt provided the pomegranate and beetroot. In the farms of upper Egypt, seder plants. No certification or approval was required to accumulate the samples because the species in question is widely distributed across the country. However, our search from the IUCN database found that Seder, Pomegranate, and Beetroots are not red-listed or classified as threatened species.

### ZnO NPs preparation

ZnO NPs were produced in four samples. One of them was made using a chemical procedure, while the final three samples were made using a green method using three different plant extracts.

#### Chemical method

0.45 M of zinc acetate prepared (4.94 g/50 ml) on the stirrer for 10 min and 0.9 M of NaOH prepared (1.8 g/50 ml) on the stirrer for 10 min and heated to 55 °C. Then zinc acetate solution is put dropwise above NaOH hot solution. The reaction was kept at 55 °C for 2 h. After that, it was kept at room temperature finally NPs washed with DW and ethanol and dried at 60 °C^36^.

#### Green procedures

##### Extract

Beetroots (B.S) extract: The local market in Egypt was the source of the fresh beetroots (beta vulgaris) used in this research. To remove dust, fresh beetroots were washed multiple times under running water. It was then rinsed numerous times with DW. After removing and peeling off the outer layers of fresh beetroots, they were sliced into little pieces. 300 g of tiny beetroot chunks in 800 mL of distilled water, heated till boiling, it was crushed to obtain uniform juice, and stored at ambient temperature until cooling. Once the extract had cooled, Wattman filter papers were used to filter it^12^.

Pomegranates (P.M) extract: Dust was removed from the pomegranates by repeatedly washing them under running water. The pomegranate was then repeatedly cleaned using DW. Cut into small pieces, 240 g of fresh pomegranate peels were heated in 1200 mL DW till boiling, and then allowed to cool at room temperature. The required extract was obtained by filtering the extract using Wattman filter papers after it had cooled^37^.

Seder extract: 140 g of recently harvested Seder leaves were properly washed under running water to get rid of any dust. The leaves were then put through several DW washes. After heating and refluxing 700 mL of DW for two hours, Seder leaves were supplementary to produce a bright yellow solution. Before, the solution was kept at room temperature and allowed to cool. The desired extract was extracted from the extract using Wattman filter sheets once it had cooled^37^.

### Green ZnO NPs preparation

**Figure 1:**
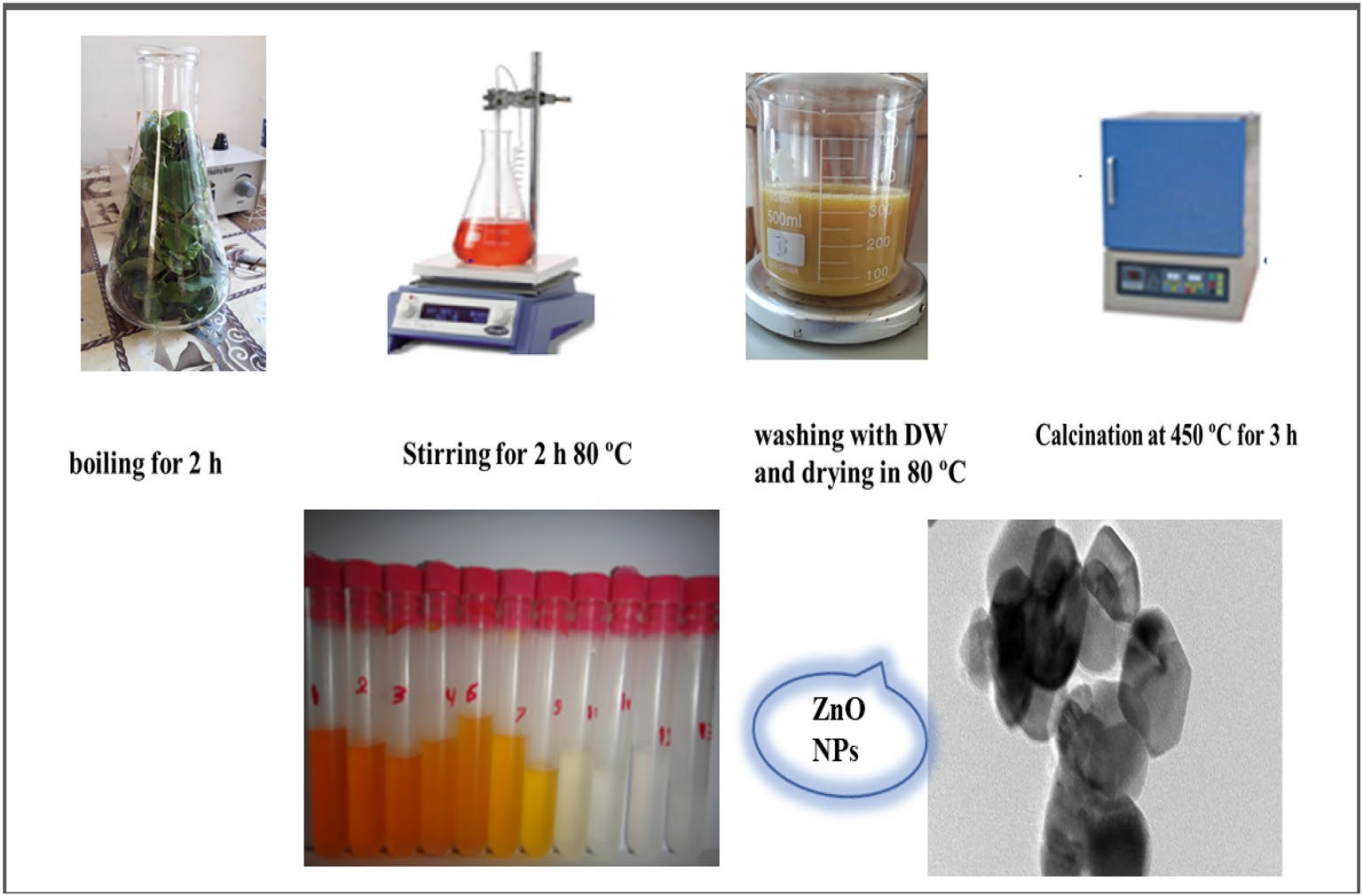
Preparation technique of ZnO nanoparticles by green method.

Gradually add 12 g zinc acetate after 30 min of stirring 600 ml of prepared extract. The response was sustained for thirty minutes with the same setup. It then reached 80 °C. Two hours were spent with the solution in this condition. Before drying at 80 °C, the particle was repeatedly cleaned with DW. Then, it was calcined for three hours at 450 °C^38^ Fig. 1.

### Photocatalytic activity

The photocatalytic activity of the materials was estimated using MO dye degradation. Open-air and room temperature were used for the trials. There were two separate experiments, one using the MO solution at 50 mg/L (PH = 7.6) and the other with the photocatalyst at 75 mg/L, which were carried out in a Pyrex beaker. A UV lamp was used to illuminate the beaker (15 W). The UV lamp was pointed at the beaker of the solution. Thirty minutes of stirring in the dark were required to achieve adsorption/desorption equilibrium. A constant stirring motion was used to keep the liquids in suspension throughout the experiment. It was also centrifuged for 15 min at 4000 rpm to separate the nanoparticles from the suspension. An Analytic Jena, Specord 200 plus UV–Vis spectrophotometer (190–1100 nm) was used to measure the MO concentrations. The absorption at 464 nm was observed. To create a calibration plot, the absorbance was linked to concentration. When the dye solution was lighted, the experiment was likewise carried out without the use of catalysts^12^.

## Characterization of the NPs

### X-ray diffraction (XRD)

For the synthesized photocatalyst NPs, X-ray diffraction (XRD) configuration data were collected at room temperature using a PANalytical diffractometer equipped with graphite monochromated CuKα radiation (λ = 1.54056 Å). 40 kV/30 mA, a step of 0.06, an interval of 4 to 89.98, and a scan rate of 2.5°/min were the operating parameters.

### High-resolution transmission electron microscope (HRTEM)

(JEOL, JEM-2100-Japan) were used to achieve the morphological studies and crystalline properties for all synthesized NPS, which offer an accelerated voltage up to 200 kv.

### Field emission scanning electron microscope (FEMSEM)

FEI Quanta 250 SEM model was manufactured by Thermo Fisher Scientific Company and used to perform dynamic in situ analysis of diverse samples in their natural state. Also used for structural and chemical analysis of metallographic specimens. Magnification up to 1,000,000× and down to a resolution of 3 nm.

### Fourier FT-IR spectra

Using a Jasco Model 4100 from Japan, which has a wavenumber range of 400–4000 cm^−1^ and a resolution of 4.0 cm^−1^, Fourier FT-IR spectra were produced for both the synthesized photocatalyst and the pomegranate extract at room temperature.

### Optical properties

With the synthesized photocatalyst at room temperature, the absorbance (A) was measured using a UV–Vis Spectrophotometer (analytic Jena, Specord 200 plus) that scanned from 190 to 1100 nm. The normal protocol was followed in the preparation of the samples by dispersing Nanopowder in DW using an ultrasonicator and putting in a 1 cm quartz covet. To study the bandgap of the photocatalyst NPs, UV–Vis spectroscopic analyses were approved. The purpose of this study was to investigate the effect of extract types on the optical band gap (E_g_) of synthesized samples. The following Eq. (1) was used to calculate E_g_^39^:

1 $$\left( {\upalpha {\text{ h}}\upupsilon } \right) = {\text{A}}\left( {{\text{h}}\upupsilon {-}{\text{E}}_{{\text{g}}} } \right)^{{\text{n}}}$$

where ***hν, A, α*** are the photon energy, transition probability factor, and absorption coefficient respectively. The energy gap (**E**_**g**_) and n are the factors that characterize the transition process. The factor n value is 1/2 for direct allowed transitions, respectively. To find the optical band gap, the linear component of the curve (αE)^2^ vs E was extrapolated.

### Surface area measurements

Were done for all prepared samples, and it added inside the results part. The surface area and pore size of NP were obtained from Brunauer–Emmett–Teller (BET) measurements using a NOVA touch 4LX analyzer (Quantachrome, [s/n:17016062702], USA). The physisorption study was carried out with N_2_ under liquid N_2_ temperature. The mean pore size distributions and total pore volume were calculated using the Barrett–Joyner–Halenda (BJH) method.

## Results and discussion

### Mechanism of plant-mediated approach

**Figure 2:**
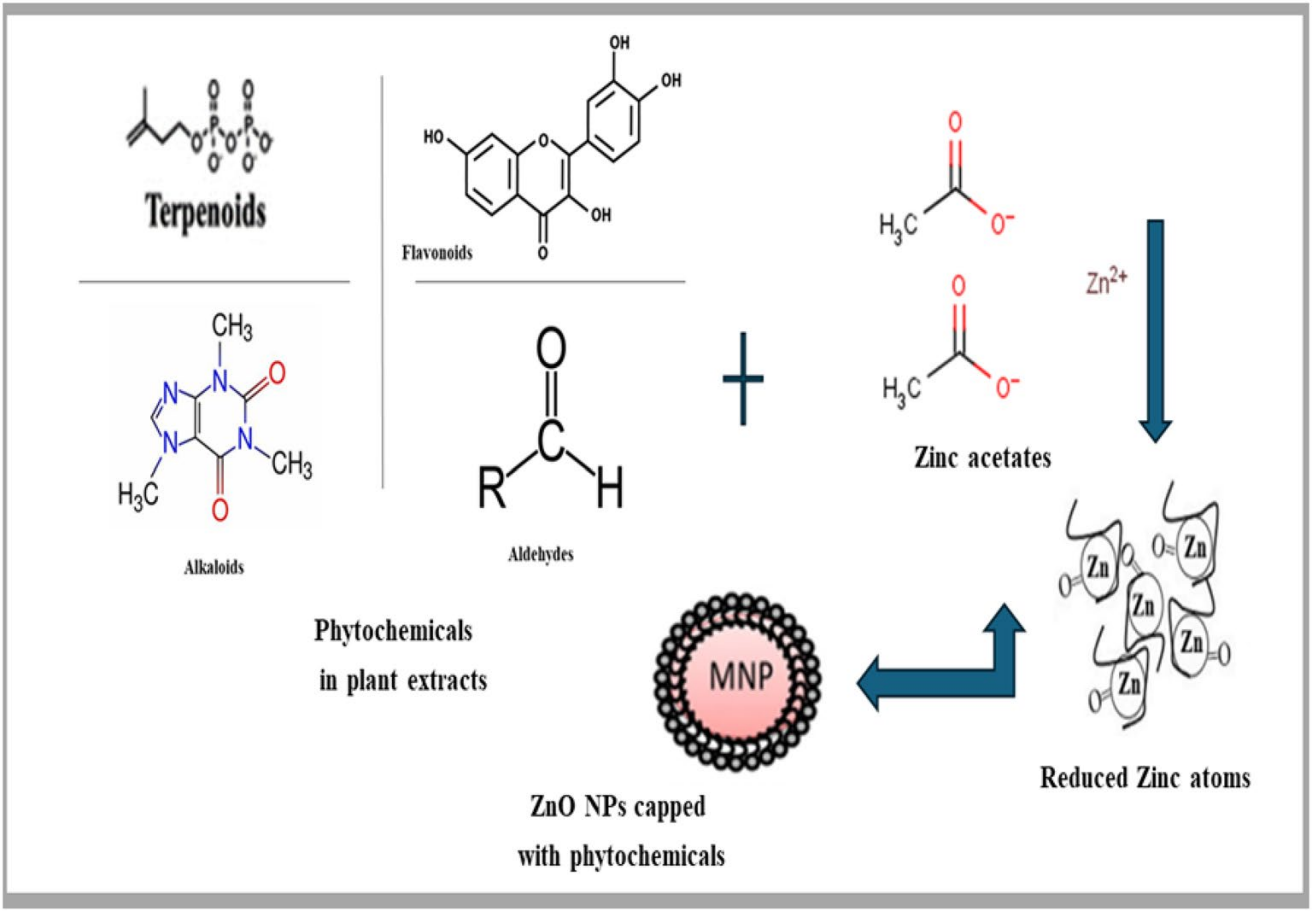
Green synthesis mechanism for fabricating ZnO nanoparticles using plant extract.

Figure 2 describes the green synthesis technique used to create ZnO NPs. The plant extract's phytochemicals can function as reducing agents to change the metal precursors into metal nanoparticles. phytochemicals can function as both stabilizing and reducing agents^40^. The reduction process has benefited from the presence of important phytochemicals such as aldehydes, flavonoids, phenolic compounds, terpenoids, and alkaloids. The quantities of these phytochemical reducing agents differ amongst plant extract varieties. Therefore, the synthesis of NPs is significantly influenced by the extract composition. The synthesis, stabilization, and quantity of nanoparticles produced are influenced by various parameters, including pH, temperature, duration of interaction, concentration of metal salts, and phytochemical characteristics of plant extract^41^. To stabilize the metal ions following reduction by plant extracts, Makarov et al. suggested encasing them in an organic coating in three phases. 1. Metal ion reduction and the nucleation of reduced metal ions are involved in the activation phase. 2. Growth phase: contributes to the stability of nanoparticles; 3. Termination phase: determines the morphology of the generated NPs^11,42^.

### XRD

**Figure 3:**
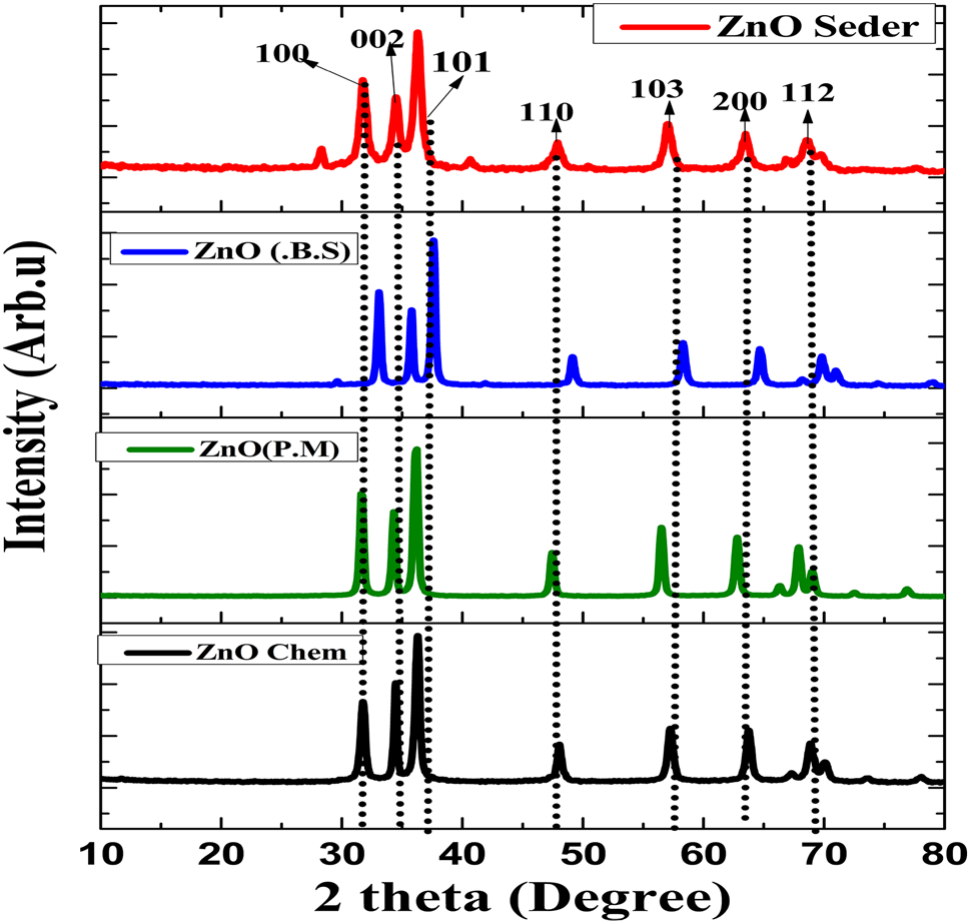
The XRD patterns of synthesized ZnO NPs with (Chem, P.M, B.S and Seder extract).

Figure 3 demonstrates the XRD patterns for the ZnO-prepared samples using different methods (chemical and green methods using different plant extracts). The diffraction peaks were observed at (100), (002), (101), (102), (110), (103), (200), (112), (201), (004), (202), (104), and (203) with a Zincite hexagonal structure [File Card No: 00-005-0664]^43^ for sample prepared using Pomegranate extract. The diffraction peaks were identified at (100), (002), (101), (102), (110), (103), (200), (112), and (201), with a Zincite hexagonal structure [File Card No: 00-005-0664] for NPs synthesized by Seder extract. The sample prepared using B.S extract exhibited typical peaks at (100), (002), (101), (102), (110), (103), (200), (112), (201), (004), and (202), indicating a Zincite hexagonal structure [File Card No: 01-078-4606] for the sample prepared using B.S extract. Characteristic peaks appeared at (100), (002), (101), (102) (110), (103), (112), and (201) with a Zincite hexagonal structure [File Card No: 00-005-0664] and (112), (004) and (202) with a Zincite hexagonal structure [File Card No: 01-078-4606] for NPs produced using a chemical process. Sharp and strong peaks indicate the high crystallinity of ZnO NPs. Based on the peak broadening in the XRD patterns, the crystallite size** D**, dislocation density, and microstrain (Eqs. 2–4) of the generated NPs can be calculated using the following relationship, as depicted in Table 1^44^.

**Table 1:** Structural lattice parameters, D, δ, ε, and E_g_ of the gained ZnO NPs using various ways (Chem, P.M extract, Seder, and B.S extract).

Method	Lattice parameter			D (nm)	δ (× 10^−4^) nm^−2^	ε (× 10^−3^)	(0 0 2)/(1 0 0)	E_g_ (eV)
	A (Aº)	C (Aº)	c/a					
P.M	3.260 ± 0.01	6.028 ± 0.02	1.847	24.5 ± 0.1	3.4 ± 0.2	17.5 ± 0.1	1.226	2.84 ± 0.05
Seder	3.258 ± 0.01	6.004 ± .0.02	1.843	22.6 ± 0.1	4.6 ± 0.2	27.0 ± 0.1	0.782	2.63 ± 0.05
B. S	3.130 ± 0.01	5.800 ± .0.02	1.850	27.3 ± 0.1	3.1 ± 0.2	16.5 ± 0.1	0.766	2.59 ± .0.05
Chem	3.256 ± 0.01	6.004 ± 0.02	1.844	30.0 ± 0.1	3.1 ± 0.2	15.7 ± 0.1	1.224	3.16 ± 0.05

2 $$\text{D}=\frac{0.94.\lambda }{\beta \cdot cos\theta }$$

3 $$\updelta =\frac{1}{D2}$$

4 $$\upvarepsilon =\frac{\beta cot \theta }{4}$$

The peak broadening and decreasing intensity of the (100) diffraction peaks indicated a decrease of the (100) crystallite size and poor crystallization of ZnO. The relative intensity ratio of (100) to (002) (I(100)/I (002)) gradually decreased^38^.The intensity ratio of (0 0 2) and (1 0 0) indicate the photocatalysis efficiency of ZnO NPs. The presence of oxygen vacancies in the produced powder has a significant impact on this ratio. In the present investigation, some defects in the structure of the prepared sample were observed, as well as a gradual increase in the (0 0 2) plane intensity. The (0 0 2) plane possesses the capacity to absorb O_2_ and OH^−^ ions as one of its inherent characteristics. Consequently, radicals are generated with strong oxidizing capabilities, playing a crucial role in the degradation of organic compounds.

### Lattice parameter

XRD data indicate that all ZnO samples prepared have a hexagonal structure. The lattice parameter is calculated using Eq. (5)^45^.

5 $$\frac{1}{{d}_{hkl}^{2}}= \frac{4}{3} \{\left(\frac{{h}^{2}+hk+{k}^{2}}{{a}^{2}}\right)+\left(\frac{{l}^{2}}{{c}^{2}}\right)\}$$

The structural lattice parameters of ZnO NPs produced by several techniques (P.M, Seder, B.S., and chemical), were illustrated in Table 1.

### Morphology of ZnO NPs

**Figure 4:**
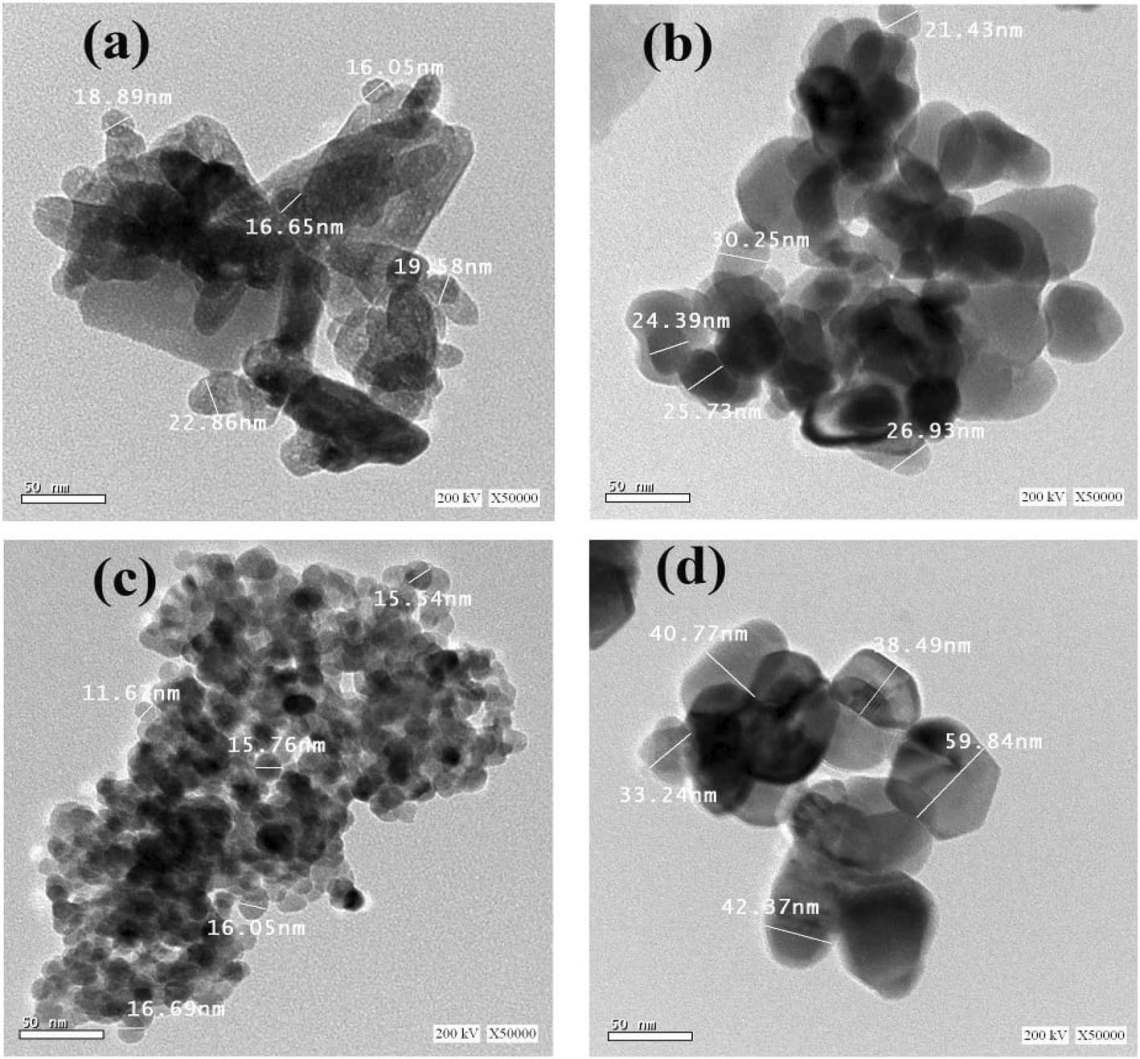
The HRTEM of the prepared ZnO NPs using (**a**) Chem, (**b**) P.M, (**c**) Seder, and (**d**) B.S methods.

HRTEM was used to determine the size and shape of the prepared nanoparticles. As demonstrated in Fig. 4a–d for ZnO-NPs prepared using Chemical (a), P.M (b), Seder (c), and B.S (d) extract., the nanostructure, spherical, and hexagonal shape for the P.M and Seder prepared samples and the hexagonal for the B.S sample were assessed. The average particle size for prepared ZnO NPs was around 19 nm for ZnO prepared with the chemical method, 27 nm for ZnO (P.M), 15 nm for ZnO (seder), and 42 nm for ZnO (B.S). The presence of aggregation can be attributed to the high surface energy of ZnO-NPs gained during preparation in aqueous media, in addition to the densification process, which leads to a confined space between particles. The chemically synthesized ZnO-NPs exist in two distinct forms, namely spherical structures, as shown in Fig. 4a.

#### FESEM

**Figure 5:**
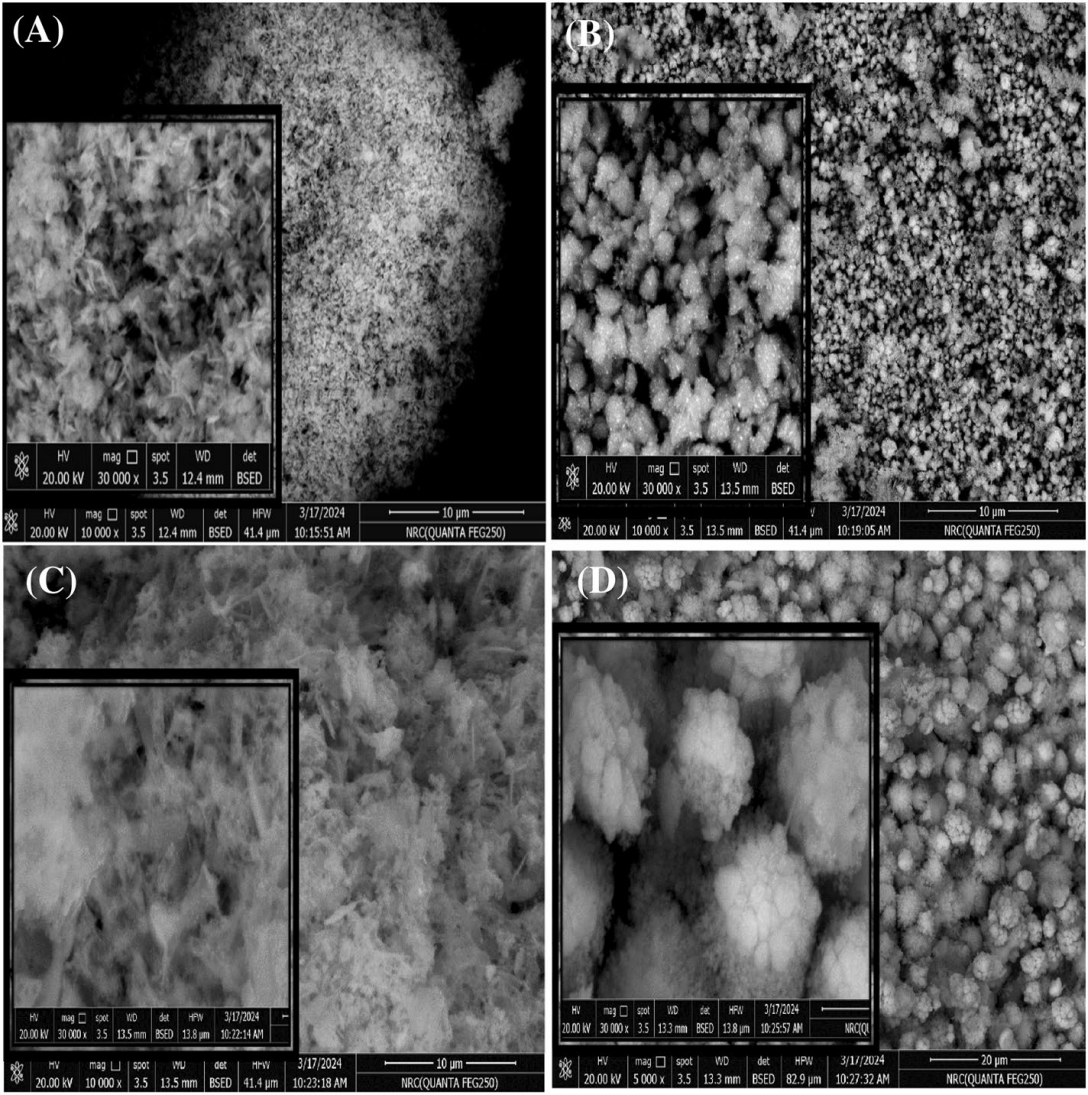
The SEM of the prepared ZnO NPs using (**a**) Chem, (**b**) B.S, (**c**) Seder, and (**d**) P.M methods.

The surface morphologies of chemical and green synthesized ZnO nanoparticles with different materials were studied by using SEM. Figure 5a–d shows the surface shape of synthesized (a) ZnO-NPs by chemical method and synthesized green ZnO-NPs prepared using (b) Beetroots (c) Seder and (d) Pomegranate extract at various resolutions 10,000× and 50,000×. All The produced products are crystalline according to detailed structural characterizations and following the confirmation of the XRD data^46,47^. The SEM images of chemically synthesized (a) ZnO show a dense collection of nanoparticles and have a nano rods-like shape with homogenous distribution. The SEM images of green synthesized (b) ZnO show homogenous nanoparticles with a spherical form while the picture of (c) ZnO shows aggregated nanoparticles with no holes and particles have a leaves flower-like shape and the picture of (d) ZnO shows a homogenous distribution nanoparticles and the shape is a uniformly and finely distributed flower^46–49^.

### FT-IR spectra

#### Extracts

**Figure 6:**
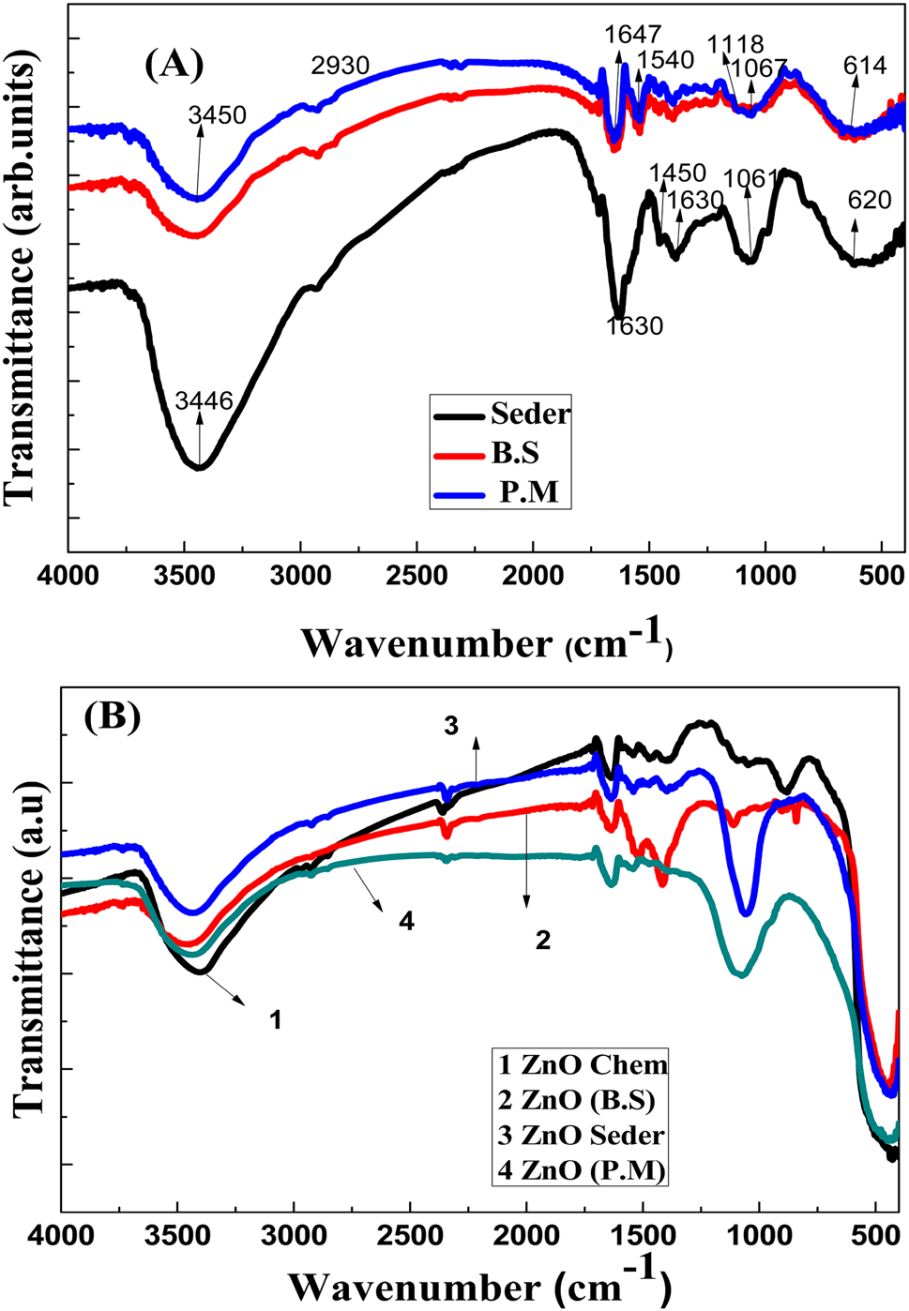
FT-IR spectra of the extracts (**A**) and (**B**) for ZnO NPs produced with different approaches (Chem, B.S, P.M, and Seder).

Stretching frequencies for the hydroxyl group (O–H) in P.M, B.S, and Seder extracts were observed at 3434, 3450, and 3446 cm^−1^ (alcohols-phenols), respectively; weak peak for the alkane group (C–H aliphatic), these stretching frequencies were 2930 cm^−1^. functional group N–H bending appeared at 1545 cm^−1^ and 1540 cm^−1^ corresponding to both P.M, and B.S, respectively, and the functional group O–H bending appeared at 1647 cm^−1^ for both P.M and B.S extracts. Additionally, the asymmetric stretching frequency emanated at 1630 for the Seder extract. Furthermore, the peaks that were observed at 1462 cm^−1^ and 1450 cm^−1^ correspond to the extracts of P.M and Seder are due to the presence of primary and secondary amines that are characteristics of proteins/enzymes and C–O stretching regions of polysaccharides and phenolic groups. Additionally, peaks represented unreacted ketone groups indicating the occurrence of flavonoids in the nanoparticles at 1340, 1390 cm^−1^ on the Seder extract, whereas P.M and B.S are indicated by at 1340 cm^−1  ^^6,7,10,50^. The S=O bond at 1060 cm^−1^ and 1061 cm^−1^ corresponds to P.M and Seder, whereas the C–O peak at 1226 cm^−1^ corresponds to P.M alone. Conversely, as shown in Fig. 6A, the C–H bend for the Seder extract was observed at 620 cm^−1^.

#### NPs

The (FT-IR) spectra for the prepared ZnO NPs are demonstrated in Fig. 6B. Alcohols showed stretching frequency for the hydroxyl group (O–H) at 3405, 3450, 3440, and 3445 cm^−1^ for ZnO NPs prepared using (chemical, B.S, Seder, and P.M). The Alkene group's (C=C) asymmetric stretching frequency came from a location at 1635 cm. N–H bending was observed at 1540, 1545, and 1560 cm^−1^ for the ZnO chemical, P.M, and Seder, respectively. The compound C=S was observed with a wavenumber of 1340 cm^−1^. The ZnO NPs generated by chemical, P.M, B.S, and Seder methods exhibited characteristic peaks below 500 cm^−1^ at 422, 435, 435, and 435 cm^−1^, respectively. These peaks are attributed to the Zn–O stretching bands^51^. From the FTIR results, the appearance of other peaks may indicate the existence of metabolites^11^ such as alkaloids, flavonoids, polyphenols, and carboxylic acid “which stayed bound to ZnO NPs despite frequent washing”. These compounds, mainly flavonoids, and other phenolics, assisted in the reduction of zinc ions to ZnO NPs. The stability of the synthesized ZnO NPs could probably be reported for the existence of free amino and carboxylic groups that have interacted with the zinc surface. Furthermore, the proteins present in the medium help in the stabilization of ZnO NPs by forming a coat, covering the ZnO NPs, and avoiding agglomeration. According to earlier research on this subject, the reduction process is caused by the phenolic groups in molecules, but the free amino and carboxylic groups may be the cause of the stability of ZnO nanoparticles^52–54^.

### Optical properties

**Figure 7:**
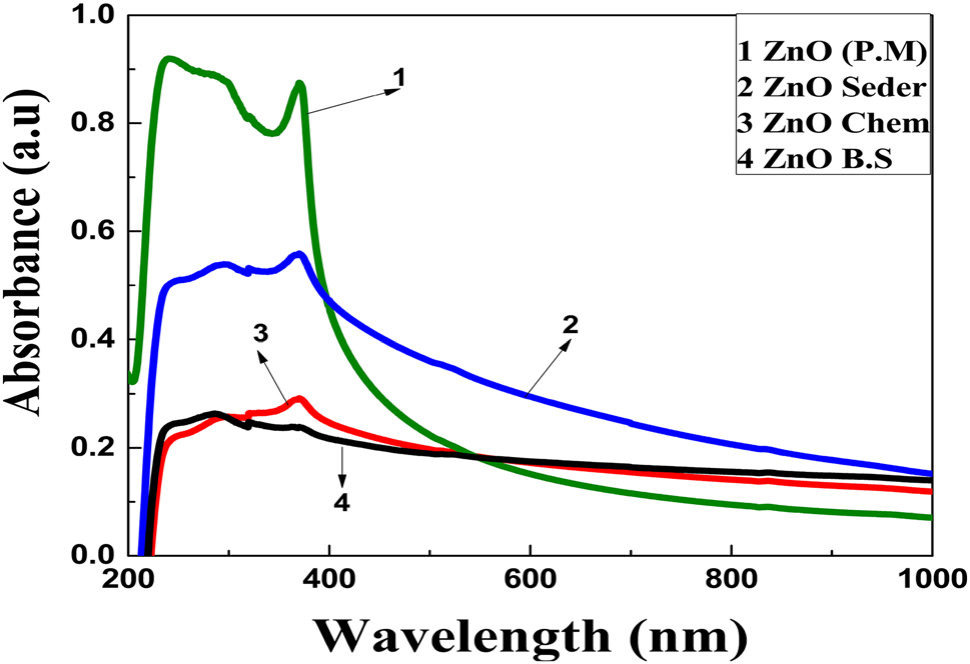
spectra of the extracts and ZnO NPs produced with different approaches (Chem, B.S, P.M, and Seder).

**Figure 8:**
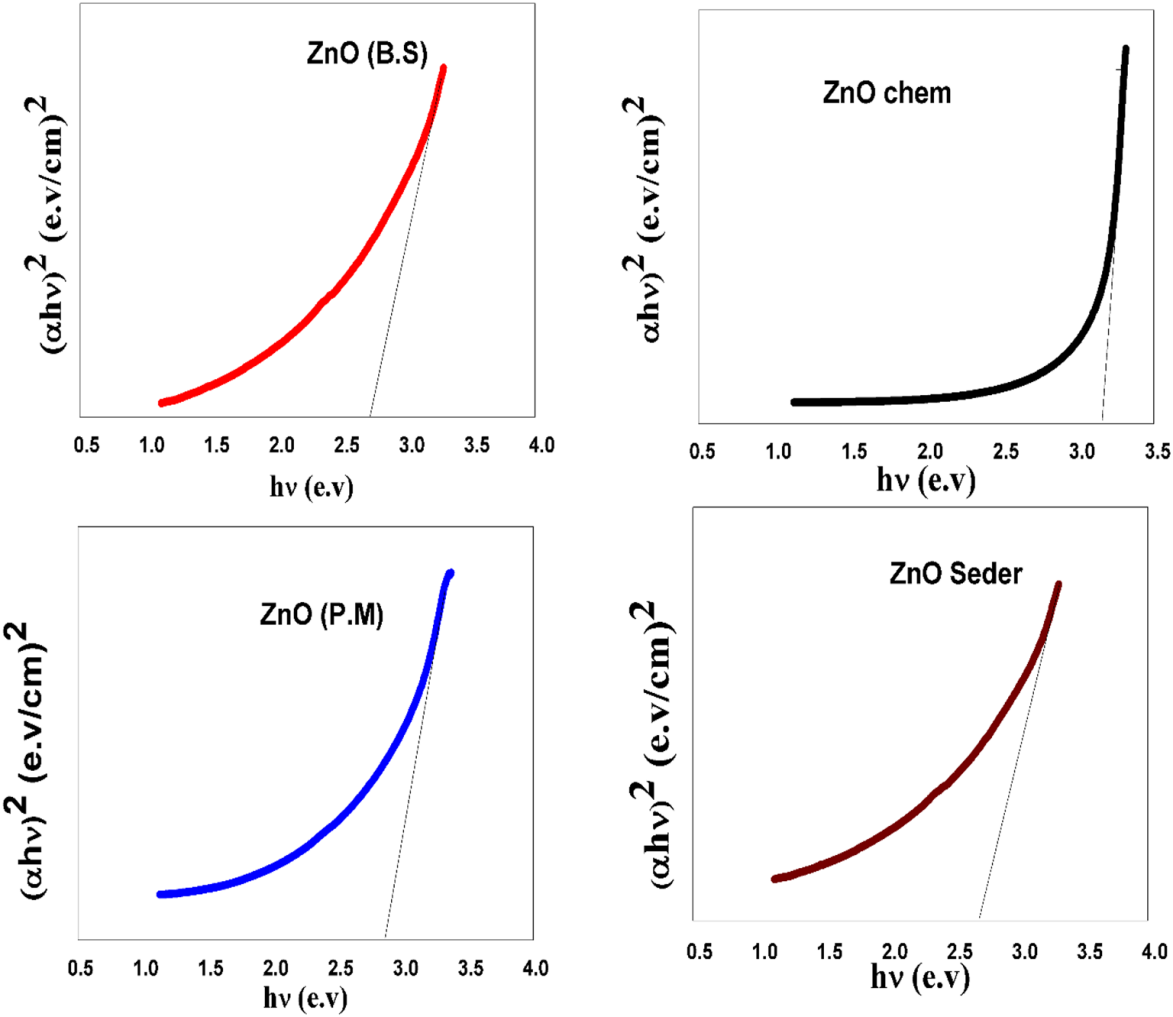
The relation between (αhν)^2^ and hν for prepared ZnO photocatalyst using (Chemical, P.M, B.S, and Seder).

In general, maximum absorption occurs at a certain wavelength due to the interaction between any materials and incident light. The absorption spectra of ZnO NPs generally depend on the parameters, such as size, shape, and temperature. With the decrease in average particle size (PS), their resonance absorption peak showed a hypochromic shift and vice versa^46^. The UV–visible absorption spectra of ZnO-NPs within a wavelength range of 200–1100 nm are shown in Fig. 7. All the samples have a strong absorption maximum below 400 nm which corresponds to the characteristic band of ZnO nanoparticles. The absence of any other absorbance peak in the spectra confirms that the synthesized products are pure ZnO NPs. The absorption maximum of the ZnO NPs produced with P.M is 295 nm, while it is 261 nm for Seder, 298 nm for B.S, and 370 nm for Chem are shifted to higher wavelengths. Furthermore, the green and blue shift is related to the decreasing crystal size of nanoparticles^47^, while the shift can be attributed to the agglomerations in samples^48,49^. The plots of (αhν)^2^ vs. E (hν) were used to determine E_g_ for the photocatalyst made of ZnO NPs made using various synthetic methods, as shown in Fig. 8. The computed optical E_g_ magnitudes of the synthesized ZnO NPs are displayed in Table 1. The kind of preparation method for the obtained NPs impacted the photocatalyst's ZnO NPs E_g_ value. The findings showed that the E_g_ values for ZnO NPs synthesized from green extracts were lower than those prepared using the chemical method. Antisemites, interstitial vacancies, and anionic or cationic vacancies originate from plant extracts that can be created during experimental activity and cause the absorption spectra to shift toward the visible region^55^. However, structural flaws or extra states concentrated inside the bandgap may be linked to the primary variations in the optical energy gap. The issues may be attributed to both changes in surface area and distinct types of oxygen vacancies. Due to the higher number of vacancies in the bandgap, more defects occur, resulting in localized states within the gap. This behavior indicates the degree of structural disorder present in these samples. These crystal defects in the produced NPs can decrease the bandgap by creating energy levels within it, acting as recombination centers^56,57^. This was attributed to doping during band gap formation, which resulted in increased defects (metal ions) from plants. Therefore, we can conclude that the visible-light absorption of ZnO could be effectively improved by introducing oxygen vacancies, and the absorption capacity was related to the concentration of oxygen vacancies.

### Specific surface area

**Figure 9:**
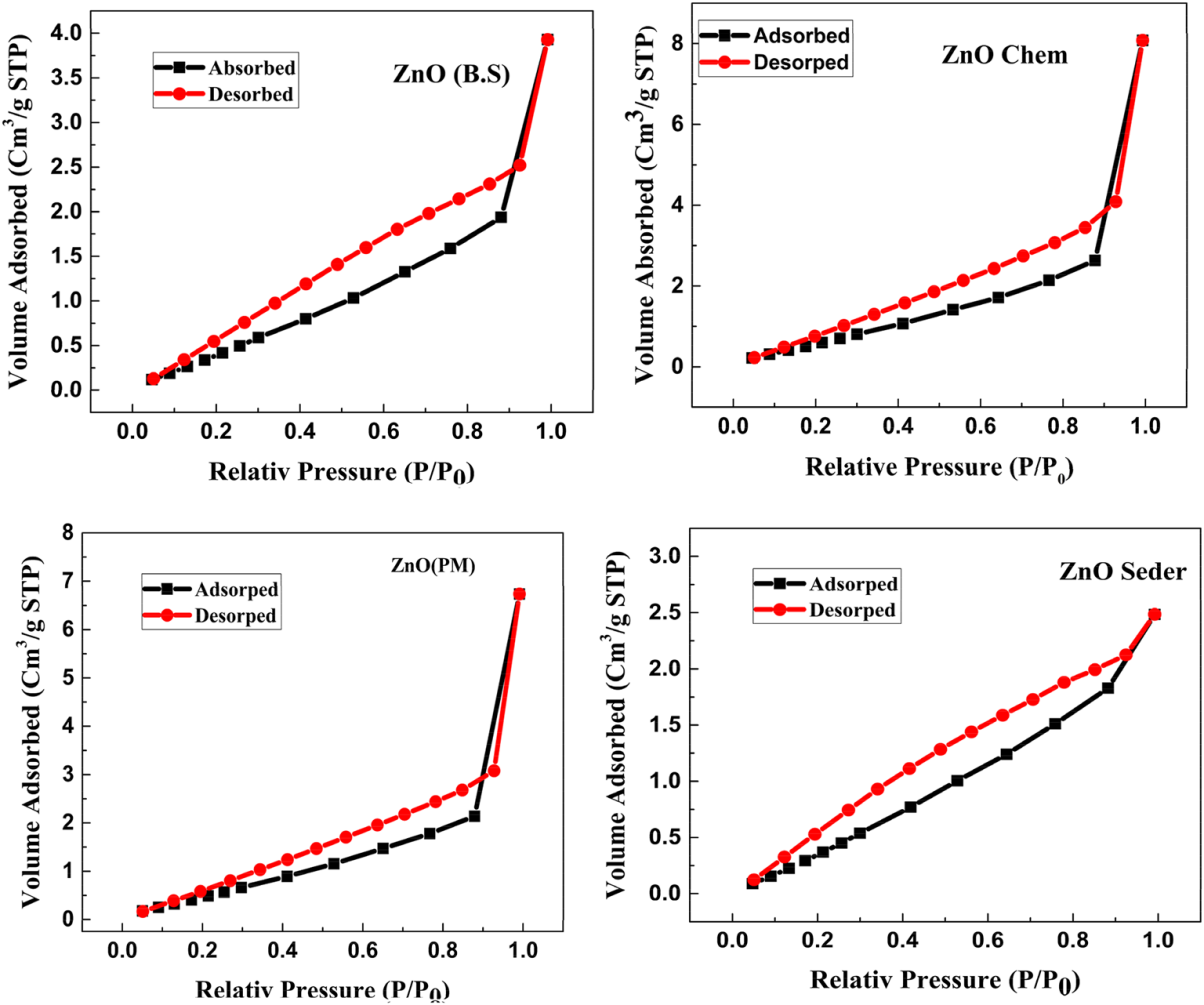
Nitrogen adsorption–desorption isotherm measured for the prepared ZnO NPs synthesized using different methods (chem. B.S, Seder, and PM).

**Figure 10:**
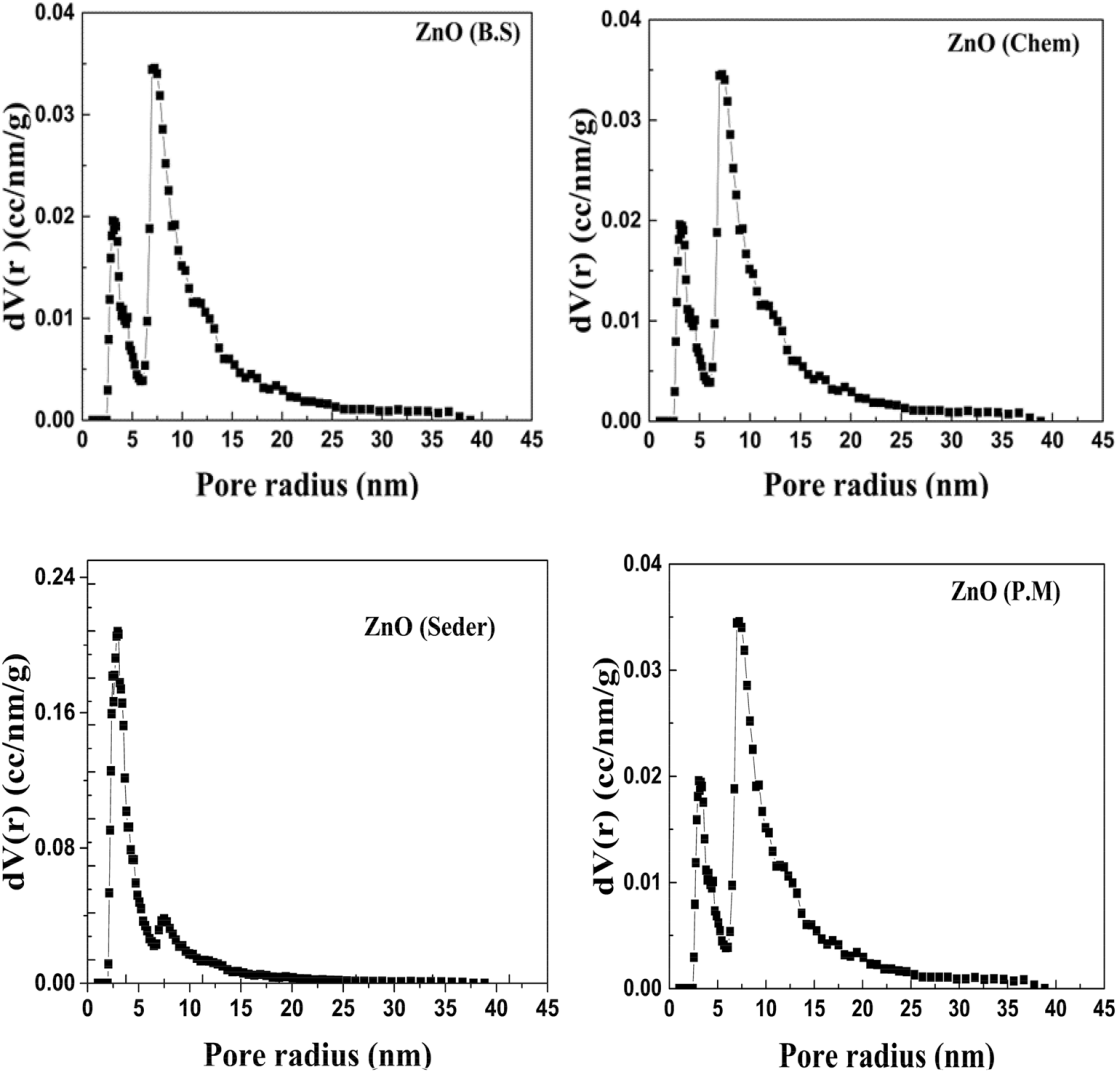
The pore size distribution (BJH curves) of the synthesized ZnO nanostructures synthesized using different methods (chem. B.S, Seder, and PM).

The BET analysis was performed to know about the surface area, pore diameter, and pore volume of the sample. The N_2_ adsorption–desorption isotherm of all prepared samples along with the Barrett–Joyner–Halenda (BJH) plot is depicted in Fig. 9. As can be observed in Fig. 8, adsorption/desorption curves of all prepared ZnO NPs show a hysteresis feature characteristic of mesoporous structures^58^. According to the International Union of Pure and Applied Chemistry (IUPAC) classifications^59^, the (Ads/Des)-isotherm is classified as IV involving an H_3 _hysteresis loop. The behavior of adsorption of N_2_ gas molecules depends on the relative pressure, such that, in regions of lower pressure, the formation of a monolayer is followed by the formation of multilayers of the adsorbed molecules in the regions of higher pressure. The total surface area obtained using the BET method, Pore radius, and Pore volume are shown in Table 2. The measured pore size and pore volume which signifies ZnO NPs as a mesoporous material. The porous materials have been classified into three categories by the International Union of Pure and Applied Chemistry (IUPAC) based on their porous sizes and diameter (d): microporosity (d < 2 nm), mesoporous (2 nm < d < 50 nm), and microporous (d > 50 nm)^60^. The BET surface areas increased with increasing N_2_ adsorption–desorption isotherms (a), pore size distributions (b) of the ZnO, and specific surface areas of the samples (c) formed under different conditions. To find some information about the pore size distributions of the green synthesized using ginger extract, Barrett–Joyner–Halenda (BJH) curves of the samples are exhibited in Fig. 10 which indicate all samples have a peak below 10 nm and the average pore radius of 1.922 nm, 1.912 nm, 1.926 nm, and 1.909 nm for ZnO (Chem), ZnO (B.S), ZnO (Seder), and ZnO (P.M) samples, respectively.

**Table 2:** Surface area, Pore radius, and pore volume the synthesized ZnO nanostructures synthesized using different ways (chem. B.S, Seder, and PM).

Sample	S Bet (m^2^/g)	Pore radius Dv (r) (nm)	Pore volume (cc/g)
ZnO chem	43.4	1.922	0.218
ZnO B.S	22.9	1.912	0.075
ZnO Seder	267.5	1.926	0.575
ZnO PM	44.6	1.909	0.225

### Water treatment

**Figure 11:**
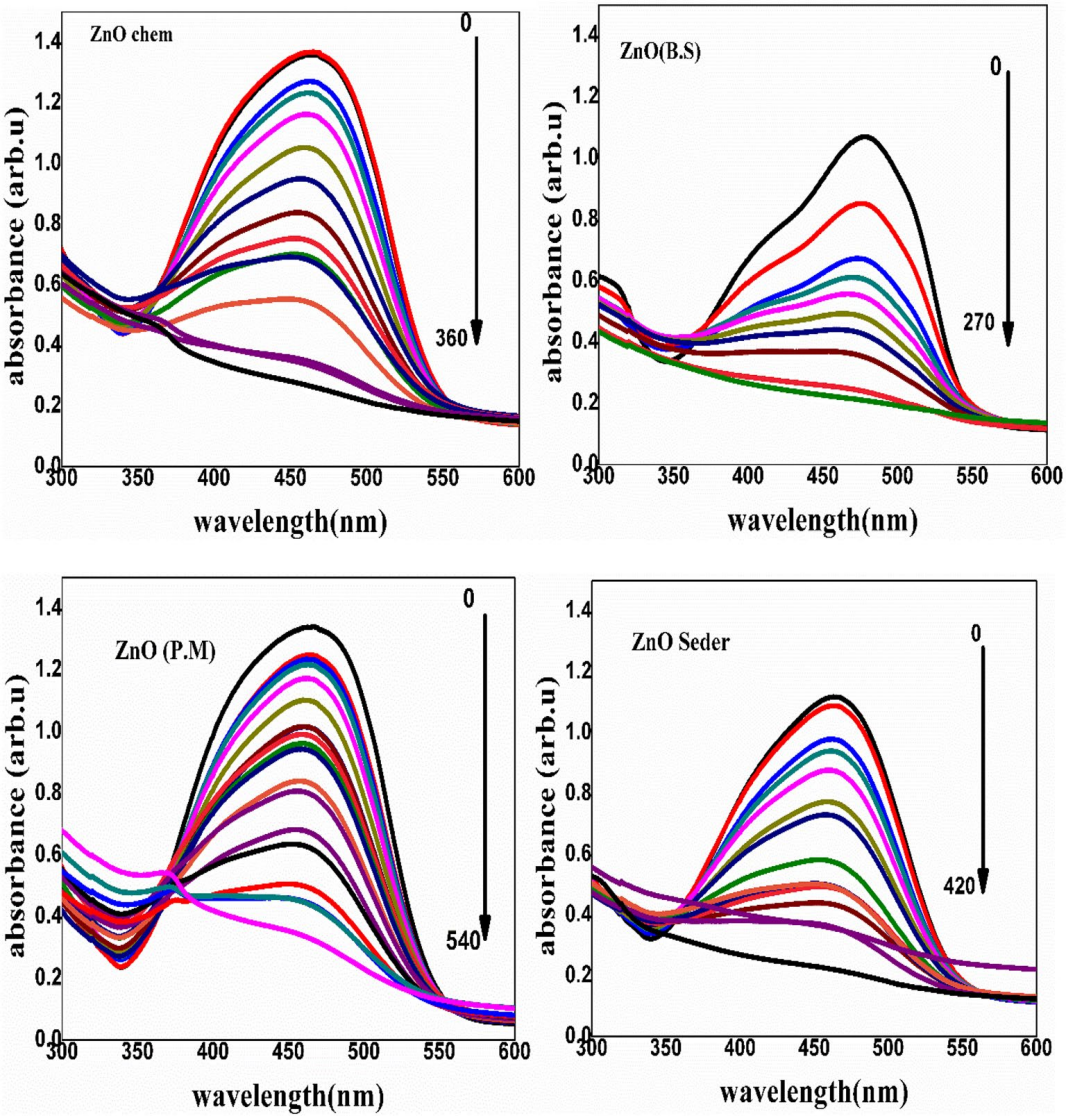
Characteristic absorbance spectra of MO through the photodegradation process with the prepared ZnO NPs.

The photocatalytic degradation process (for ZnO-NPs) was detailed in several references^3,37^, summarized in Fig. 11. According to previous work^57^, the hydroxyl radical (called Reactive Oxygen Species—ROS) represents a powerful oxidizing source that can attack organic pollutants. The e/h recombination is the primary issue during the catalysis process. In addition, the presence of defects in ZnO (oxygen vacancies in the band gap, which causes localized states to emerge within the gap) causes inhabitation for this recombination through the trapping process, leading to a high generation of ROS^56^. The photocatalytic performance of the prepared ZnO-NPs is indicated by changes in the intensity of the characteristic absorption peak with exposure time. With UV irradiation, about 464 nm was the highest absorption peak. After 270 min (B.S), 360 min (ZnO Chem), 420 min (Seder), and 540 (P.M), after equilibrium adsorption.

**Figure 12:**
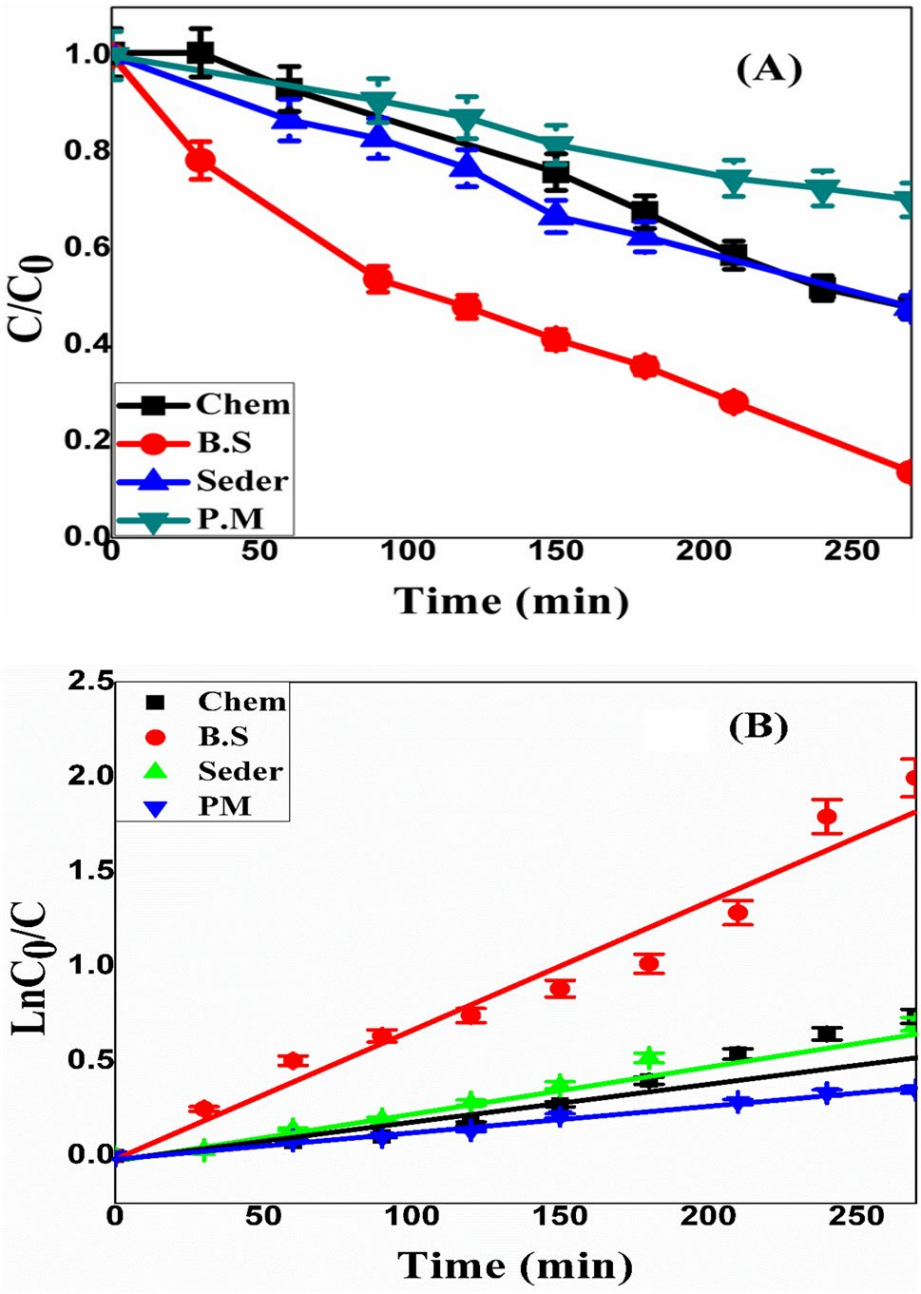
The rate of MO degradation with UV (**a**), and (**b**) ln (C/C0) as a function of exposure time For the ZnO NPs that were produced using different preparation methods (Chem, P.M, B.S., and Seder).

The results of the two empty experiments showed that MO could not undergo degradation in the absence of a photocatalyst or UV radiation. Figure 12 displays the photocatalytic performance of the produced ZnO-NPs with different methods. It is evident that green approaches were used to create ZnO-NPs exhibit more catalytic activity than the chemical method. The degradation efficiency (η) of each sample was calculated according to Eq. (6).

6 $${\upeta \%}=\frac{C-C0}{C0}\times 100$$

**C**_**0**_ represents the MO solution’s initial concentration (before treatment), and **C** represents the concentration (after time **t**) in mg/L, respectively. The pseudo-first-order kinetics of MO degradation are followed (Fig. 8). Equations (7–9) were also used to determine the kinetic rate constant (**K**) and half-time **t**_**1/2**_^61,62^.

7 $$-\frac{dCo}{dt}=\text{KCo}$$

8 $$\mathit{ln}\left(\frac{C0}{C}\right)=\text{Kt}$$

9 $${\text{t}}_{{1/2}} = \frac{{ln2}}{K}$$

**Figure 13:**
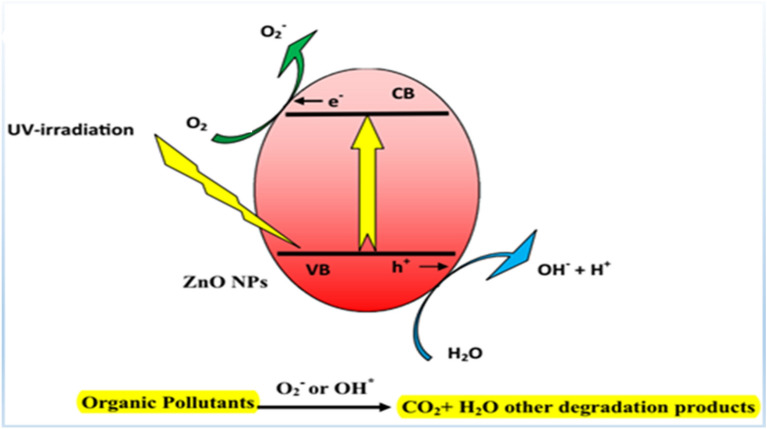
ZnO Mechanism in photocatalytic degradation of organic compounds.

The degradation efficiency for all prepared ZnO-NPs was calculated after 270 min and tabulated in Table 3. The redox reaction occurring during photocatalysis is shown in Fig. 13. As a result, the mechanism of redox reaction-mediated photodegradation of organic molecules in the presence of radiation can be summed up as follows Eqs. (10–20)^63–65^:

10 $${\text{ZnO}} + {\text{h}}\upnu \to {\text{ZnO}}({\text{e}}_{{({\text{CB}})}}^{ - } ) + ({\text{h}}_{{({\text{V}}.{\text{B}})}}^{ + } )$$

11 $${\text{ZnO}}\left( {{\text{h}}^{ + }_{{({\text{V}}.{\text{B}})}} } \right) + {\text{H}}_{2} {\text{O}} \to {\text{ZnO}} + {\text{H}}^{ + } + {\text{OH}}^{ \cdot }$$

12 $${\text{ZnO}}\left( {{\text{h}}^{ + }_{{({\text{V}}.{\text{B}})}} } \right) + {\text{OH}}^{ - } \to {\text{ZnO}} + {\text{OH}}^{ \cdot }$$

13 $${\text{ZnO}}\left( {{\text{e}}^{ - }_{{({\text{CB}})}} } \right) + {\text{O}}_{2} \to {\text{ZnO}} + {\text{O}}_{2}^{ \cdot - }$$

14 $${\text{O}}_{2}^{ \cdot - } + {\text{H}}^{ + } \to {\text{HO}}_{2}^{ \cdot }$$

15 $${\text{HO}}_{2}^{ \cdot } + {\text{HO}}_{2}^{ \cdot } \to {\text{H}}_{2} {\text{O}}_{2} + {\text{O}}_{2}$$

16 $${\text{ZnO}}\left( {{\text{e}}^{ - }_{{({\text{CB}})}} } \right) + {\text{H}}_{2} {\text{O}}_{2} \to {\text{OH}}^{ \cdot } + {\text{OH}}^{ - }$$

17 $${\text{H}}_{2} {\text{O}}_{2} + {\text{O}}_{2}^{ \cdot - } \to {\text{OH}}^{ \cdot } + {\text{OH}}^{ - } + {\text{O}}_{2}$$

18 $${\text{H}}_{2} {\text{O}}_{2} + {\text{h}}\upnu \to 2{\text{OH}} \cdot$$

19 $${\text{Organic pollutants}} + {\text{OH}} \cdot \to {\text{Intermediates}}$$

20 $${\text{Intermediates}} \to {\text{CO}}_{2} + {\text{H}}_{2} {\text{O}}$$

**Table 3:** K pseudo-first-order rate constant for ZnO NPs photocatalyst.

Method	K (min^−1^)	t_1/2_ (min)	R^2^	Standard error	η %
ZnO (B.S)	0.0070	99.02	0.95	0.0005	87 ± 0.5
ZnO (P.M)	0.0010	693.15	0.97	0.0001	30 ± 0.5
ZnO Seder	0.0028	247.56	0.98	0.0001	52 ± 0.5
ZnO Chem	0.0029	239.02	0.97	0.0003	53 ± 0.5

It is essential to consider that various factors, such as particle size, energy band gap, phase, carrier lifetime, catalytic surface properties, and the kinetics of molecule adsorption and desorption, can affect the rate at which a photocatalytic reaction occurs. The photocatalytic activity of ZnO increases when it is synthesized from B.S. The presence of metal ions in the material leads to a decrease in recombination processes, an improvement in carrier lifetimes, and an increase in photocatalytic activity. This is achieved by the metal ions acting as trap centers in the band gap and catching electrons^54^. The presence of excessive amounts of defects in ZnO-NPs prepared using the green method represents the primary source of increasing the degradation efficiency of such powders. This finding can be attributed to the decreases in the optical band gap due to the presence of these defects. The presence of such defects is reflected in the ratio intensities of (0 0 2) and (1 0 0) planes. The drop in this intensity ratio was linked to the emergence of a confined energy state, which lowered the energy gap (E_g_) and prevented the recombination process. Consequently, this will result in a rise in the catalytic activity.

The change in this ratio was ascribed to the polar character of the (0 0 2) plane, as stated in the literature. They ascribed this ratio to the polar characteristic of the (0 0 2) plane. It can adsorb O_2_ and OH^−^ ions to produce high oxidizing radicals, which play a crucial part in the degradation process of organic compounds. The photocatalysis is more catalytically effective when oxygen vacancies are present.

Oxygen vacancies function as electron acceptors, but interstitial oxygen enhances the number of trap sites for photo-generated holes, hence impeding the recombination process of electron–hole pairs of (e^−^ − h^+^). Furthermore, the catalytic efficiency has experienced a significant rise of 46–48% due to enhanced light absorption and efficient charge transfer facilitated by the inter-band gap states^66–68^.

As shown in Table 4, a comparison was made between the photocatalyst utilized in this study and other coupled nanoparticle photocatalysts that were published in earlier reports. The results indicate that green ZnO NPs have outstanding properties, demonstrating that this high-performance photocatalyst can revolutionize water treatment.

**Table 4:** Comparison of performance of green-prepared ZnO photocatalyst published in previous studies.

Photocatalyst	Extract	E_g_ (e.V)	Light	Efficiency	Pollutant	Refs.
ZnO (quasi-hexagonal)	Leaf of Alchornea laxiflora	2.50–3.67	Sunlight 60 min	87%	Congo red (CR) dye (1.5 mg/L)	^62^
ZnO (spherical and hexagonal)	Pullulan	3.3	UV 120 min	85.7% and 96.8% for AMX and PCT	amoxicillin (AMX) and paracetamol (PCT) 30 ppm	^69^
ZnO (nanorods)	garlic bulbs (Allium Sativum)	3	solar light 150 min	96%	methylene blue (MB) 10 ppm	^70^
ZnO (Spherical)	lime juice	3.13	(UV) (75 min) and sunlight (60 min)	98.8%, 94.8%, 98.5% 98.8% for MB, MO, RhB PRA under solar, 92.6%, 92.5%, 93.2%, 92.9% under UV	MB, MO, RhB and PRA dyes	^71^
ZnO (flower like shape)	Euphorbia sanguinea	2.72–4.37	Solar radiation 60 min	92%	Malachite green dye (1.5 mg/L)	^72^
ZnO (Spherical and hexagonal shape)	g Actinidia deliciosa (kiwi) fruit peel	3.06	U.V 120 min	96.3%	p-bromophenol 15 mg/L	^73^
ZnO (semi-spherical morphology)	Justicia spicigera	3.3	UV 120 min	90%	methylene blue (MB) 15mg/L	^74^
ZnO (Spherical)	(Lantana camara flowers)	3.56	UV 80 min	(98%)	methylene blue (MB) 20 mg/L	^75^
ZnO (Rod shaped)	Ruellia tuberosa	–-	Sunlight 150 min	94% and 92% of (MB) and (MG)	methylene blue (MB) and malachite green (MG) (10 mg/L)	^76^
ZnO (Sphere and hexagonal)	pomegranate, beetroot roots, and seder	2.84, 2.63,and 2.59	UV 270 min	32, 87, and 52%	Methyl orange MO (50mg/L)	Current study

**Figure 14:**
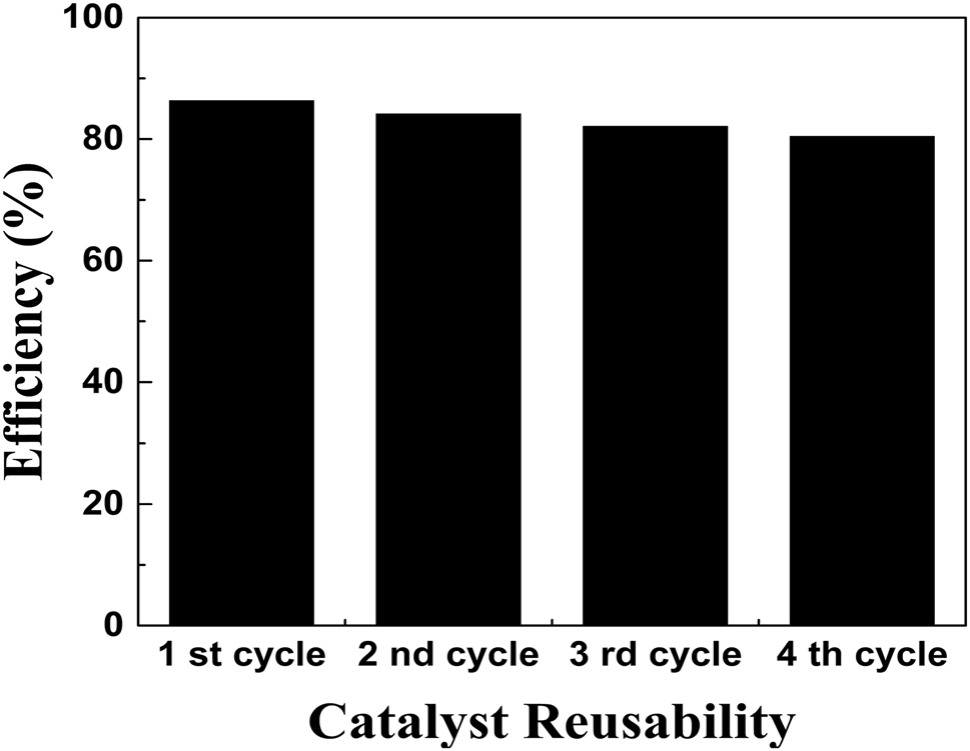
The recycle test of ZnO (B.S) under UV irradiation against MO dyes.

To estimate the durability of the ZnO NPs, four cyclic experiments were also conducted by the photocatalytic degradation of MO dyes for the best ZnO sample that was prepared using (B.S) extract in the same condition, an actual experiment was repeated for 4 cycles. The results of the photocatalysis cycle tests in Fig. 14 confirm that the photocatalytic property of the ZnO (B.S is not significantly lowered, and the photodegradation efficiency achieves 80% after four cycle runs. This demonstrates that ZnO NPs are excellent reusable and recyclable photocatalysts for the photo-degradation efficiency of MO dyes, which may be potential candidates for wastewater treatment applications.

## Conclusion

This paper examined the photocatalytic activity of ZnO NPs prepared using the chemical and green method. The results indicated that ZnO NPs prepared utilizing the green method are cost-effective, eco-friendly, and highly efficient catalysts for a broad range of photocatalytic applications, particularly for organic molecules. The properties of the synthesized ZnO nanoparticles were examined using XRD, FESEM, Surface area, FTIR, UV- Vis and TEM techniques. The plant extract was analyzed using FTIR to determine the presence of specific biomolecules involved in the synthesis of ZnO nanoparticles. The MO solution was subjected to photodegradation in UV light using both types of ZnO-NPs. The abundance of excessive defects in the ZnO-NPs generated using the green method is the primary factor contributing to the improvement of degrading efficiency. The photocatalytic activity of the produced nanoparticles exhibited an apparent pseudo-first-order kinetics trend. The study suggests that ZnO-NPs generated using plant extracts exhibit superior efficiency compared to ZnO-NPs synthesized using chemical methods. The most effective ZnO NPs, synthesized using Beetroots, exhibited a degradation efficiency of 87 ± 0.5% with a kinetic rate constant of 0.007 min^−1^ as it has the lowest E_g_, and higher in oxygen vacancies, which enhances their photocatalytic efficiency. The potential utility of our research lies in its application within the water treatment industry, specifically as an effective technique for modifying ZnO photocatalysts.

## Data Availability

The datasets used and/or analyzed during the current study available from the corresponding author on reasonable request.
